# Pioneering COVID-19 Pandemic Partnerships: Federally Qualified Health Centers and Geriatric Workforce Enhancement Programs Work Together to Care for Diverse Underserved Older Adults

**DOI:** 10.1177/23337214221122523

**Published:** 2022-09-06

**Authors:** Lisa Gibbs, Julie Rousseau, Nina Tumosa, Roopali Gupta, Bonnie Olsen, Anna Faul, Jennifer Reichstadt, Samantha Cotton, Theresa Sivers-Teixiera, Neika Saville, Jung-Ah Lee

**Affiliations:** 1 University of California, Irvine, USA; 2 U.S. Bureau of Health Workforce, Rockville, MD, USA; 3 University of California, San Diego, La Jolla, USA; 4 University of Southern California, Los Angeles, USA; 5 University of Louisville, KY, USA

**Keywords:** Geriatric Workforce Enhancement Programs, Federally Qualified Health Centers, diverse older adults, COVID-19 pandemic, challenges

## Abstract

**Background:** The COVID-19 pandemic disproportionately affected populations served by Federally Qualified Health Centers (FQHCs), with high morbidity and mortality rates in ethnic minority older adults. In response to this pandemic, academic geriatric medicine teams through federally funded Geriatric Workforce Enhancement Program (GWEP) with FQHC partnership implemented new initiatives to improve the care for vulnerable older adults. **Objectives:** To describe how four FQHC/GWEP teams collaborated in caring for diverse communities of older adults during the pandemic. **Methods:** Four GWEPs have addressed pandemic response efforts with their respective FQHC partners. These collaborations to meet the increasing numbers of older adults seeking services, and the rising disparities exacerbated during the pandemic are delineated. **Results:** FQHC/GWEP partnerships enabled access to care, whether in-person or virtually to serve unmet needs of underserved older adults during the pandemic. Partnerships promoted COVID-19 education, testing, and vaccinations. Most FQHCs faced severe staffing shortages, and the digital divide challenged patients with barriers. GWEPs provided direct care, created educational materials, and developed telehealth programs. These partnerships addressed social determinants of health gaps caused by the pandemic. **Conclusion:** The findings demonstrate that strong partnerships between GWEPs and FQHCs mitigate health inequities for vulnerable ethnic minority and rural older adults during pandemic crises.

The United States (U.S.) Census Bureau projects a rise in the adult population age 65 and older from 54.1 million to 80.4 million by 2040, and another dramatic increase to 94.7 million by 2060 (Administration for Community Living, 2021). The U.S. faces a crisis with a rapidly aging population, yet a shortfall of geriatric health providers to support diverse older adults; the exodus of approximately 20% of healthcare workers during the pandemic will only accelerate this workforce shortage (Levine, 2021). To meet these challenges, four Geriatric Workforce Enhancement Programs (GWEP), funded by the Health Resources and Services Administration (HRSA), a federal agency within the Department of Health and Human Services (HHS), have strengthened partnerships with local Federally Qualified Health Centers (FQHCs). The pandemic disproportionately affected the populations that FQHCs serve, causing higher morbidity and mortality for diverse ethnic older adults. The GWEP program leaders met over several sessions to compare and contrast their FQHC collaborations during the pandemic. GWEP leaders sought to bolster FQHCs’ responses to the pandemic; to strengthen emerging geriatric-focused healthcare programs; and to create pipelines for future healthcare professions able to care for the increasing numbers of older adults seeking services at FQHCs.

## The Geriatric Workforce Enhancement Programs

There are currently 48 GWEPs supported by HHS/HRSA in 35 of the 50 states and 2 territories. Collectively, the GWEPs leverage geriatric expertise to educate and train the primary care workforce to improve care for older adults, tailoring this training based on community-specific needs, promoting age-friendly health systems and dementia-friendly communities, and addressing healthcare gaps and social determinants of health. GWEPs also create pipelines of future healthcare professionals and deliver community-based programming to patients, families, and caregivers. The GWEPs promote partnerships between academia, primary care delivery systems, including FQHCs, and community-based organizations, such as Area Agencies on Aging (HRSA, n.d.a, n.d.b, n.d.c).

## GWEP Partnerships With FQHCs

GWEPs address the startling increase in impoverished older adults and the disproportionate burden of disease and social determinants of health faced by older adults. Approximately, 14.1%, or 7.2 million individuals over the age of 65 are living at or below the federal poverty level (Cubanski et al., 2018). To address these disparities, HRSA provided a funding preference under PHS Act, Section 805 for GWEP applicants with projects that substantially benefit rural or underserved populations. For many GWEP programs, this promoted partnerships with FQHCs: FQHCs deliver comprehensive, culturally competent, high-quality primary health care services to the nation’s most vulnerable individuals and families (HRSA, n.d.a). In the U.S., HRSA-funded Health Centers serve one in three people living in poverty, one in five uninsured, one in five rural residents and approximately 3 million adults over the age of 65 (HRSA, n.d.b). While the current percentage of patients over 65 who receive care at FQHCs is usually <10%, the FQHCs are expected to play an ever-increasing role in the primary care safety net for older adults. Of the 48 GWEP programs, 32 have partnerships with a total of 190 FQHCs. Through these partnerships, GWEP programs are working to reduce disparities and improve healthcare outcomes by developing Age Friendly Health Systems, with a focus on mentation, mobility, medications, and what matters to patients (four M’s), in underserved and impoverished communities (Institute of Healthcare Improvement [IHI], 2020).

## Populations Served

Four GWEPs, three in California, and one in Kentucky, have built strong partnerships with nine local FQHCs, that serve diverse communities and cover a wide range of urban and rural underserved populations. These nine FQHC partners serve their surrounding communities with a shared catchment of approximately 12% to 15% uninsured and 97% to 99% with incomes under 200% of the federal poverty level (FPL). Several FQHCs serving urban and rural populations have a high proportion, 50% to 75%, of patients identifying as Latinx. Two sites are located within Asian communities, Vietnamese and Korean, serving 75% of those identifying as Asian. Another FQHC is the only primary care clinic specializing in LGBTQ for a large urban setting, serving over 870 older adults in that community. The rural-located FQHCs serve high rates of migrant farm workers and patients experiencing homelessness. Another FQHC focuses on patients with poor access to healthcare in a largely rural setting, serving a predominantly white, 10% uninsured population. Older adult populations served across all partner FQHC clinics range from 6-16%.

## COVID-19 Pandemic Challenges and Responses

In the U.S. 75% (approximately 600,000) of all COVID-19 related deaths to date have occurred in persons 65 and older: COVID-19 is now the third leading cause of death in this age group (*New York Times*, 2021). The pandemic morbidity and mortality statistics highlight healthcare disparities, which disproportionately affect older adults of color that FQHCs serve (Centers for Disease Control and Prevention [CDC], 2021; Guerrero & Wallace, 2021). Because FQHC clients were more vulnerable than average to COVID-19, FQHCs quickly pivoted to serve their diverse and underserved older adult communities as the pandemic progressed, first by ensuring access to care, whether in person or virtual, and secondly, by becoming centers for testing, COVID-19 care, and vaccinations. Because GWEPs and affiliated FQHCs had been working together for 2 to 7 years prior to the pandemic, GWEPs were able to offer support in unique ways. The GWEP teams have identified three key challenges exacerbated in the pandemic, and the efforts that the GWEP-FQHC partnerships undertook to meet these needs.

## Challenge 1: Impact of the Pandemic on Clinical Resources and Access to Care

In addition to dealing with long-standing challenges in recruiting and retention of frontline healthcare workers across the FQHC staff (California Health Care Foundation [CHCF], 2021), the COVID pandemic caused staff members to become ill, quarantine, and provide care to their own families. These challenges resulted in high staff turn-over and workforce shortages. Clinics experienced high no-show rates for visits and needed to learn how to support and provide telehealth audio and video encounters, while remaining open for patients needing in-person visits or facing barriers to telehealth use. One study reported that overall FQHC utilization across the U.S. decreased 23% (Simon et al., 2021). Local FQHCs became focal points for testing sites and vaccine campaigns, stretching existing resources and further limiting the availability of preventative and chronic care (Simon et al., 2021).

To supplement the overstretched FQHC staff, GWEPs partnered to provide education and outreach: GWEP students recorded COVID-19 education videos in English, Spanish, and Vietnamese for distribution in the FQHC communities. After the vaccines became available, vaccine delivery and addressing hesitancy became a new challenge, as clinics cared for hard-to-reach communities suffering from high COVID morbidity and mortality. The disparities in care access and pandemic-related mental health issues for Asian Americans at one San Francisco FQHC attest to the need for support (Quach et al., 2021). A novel program, Love Our Vulnerable and Elderly (LOVE), united nonprofits organizations and a GWEP to address needs of vulnerable Asian Americans in Southern California (Constante, 2021). In one FQHC, a GWEP-funded, Spanish-speaking geriatrician continued to care for older patients throughout the pandemic, addressing specific needs of Latinx older patients, including dementia and caregiver support.

## Challenge 2: Technology Challenges

At the height of the pandemic in 2020, older adults were encouraged to stay at home as the safest option, and many patients were unwilling to leave their homes for routine healthcare appointments. Although academic medical centers and FQHCs continued to see those in need of in-person visits, telehealth became a preferred way to safely deliver primary care. As healthcare providers embraced telehealth as the safest way to see patients at the height of the pandemic, the digital divide for older adults became a social determinant in and of itself, separating those who could safely access healthcare via telehealth technology and those who could not. The pandemic necessitated a pivot to telehealth delivered virtual visits, with some partner FQHCs reporting a drop of 70% to 80% for in-person visits. In fact, HRSA reports that FQHCs provided over 28.5 million virtual visits, an increase of about 6,000% from 2019. In reviewing reporting data for California in 2020, across 175 FQHCs approximately 52% of all visits with providers were conducted virtually, for a total of 5,763,887 virtual visits. (HRSA, n.d.a, n.d.b, n.d.c). All partnerships report that many patients faced low technology literacy and limited access to devices and broadband service. Even if households had access to a computer or smartphone, some geographic regions had poor or no bandwidth and connectivity.

The ability for individual FQHC sites to transition to telehealth varied. Some transitioned quickly, while some reported a slow process dependent on a limited number of tablets, laptops, and software to share among the providers. Other sites resorted to telephone encounters due to the digital divide and the clinics’ inability to invest in a telehealth platform; this trend mirrors the national FQHC reporting on the reliance on telephone visits (CHCF, 2021). Even when some organizations moved quickly to adapt to telehealth visits, provider training lagged due to limited hardware and software availability.

To help address these challenges, one GWEP provided patients with tablets and hotspots for connectivity. Some GWEPs were able to offer access to additional telehealth equipment and training—both to providers and to patients, for whom digital literacy was low. In another site, a GWEP-related CARES Act supplemental fund supported a telehealth pilot for older adults, which benefited the FQHC site through electronic medical record data integration and training. FQHC partners noted that, as a result of the pandemic and the shift to virtual services, patients were more receptive than before to receiving services via phone or video. At the height of the pandemic, patients requiring in-person visits continued to be seen at the FQHCs and academic medical centers; these community centers remained open to meet the needs of their patients, including those suffering with COVID. Many patients understood the need to isolate for their own safety and the safety of their families; thus, they stated a preference for telehealth at the height of the pandemic and continue to embrace telehealth during widespread COVID surges.

## Challenge 3: New Social Determinants of Health Gaps

With the pandemic, needs assessments of social determinants of health (SDOH) became more critical. Community-based organizations were forced to curtail services or provide service remotely, decreasing access for SDOH and socialization needs. Lack of access to resources such as transportation and nutrition were increased. FQHC clinics struggled to continue their ancillary services, such as free legal, tax, and other social service enrollment (except for Medicaid) assistance, due to the pandemic. Limitations from both the community service and FQHC perspectives not only worsened, but created new SDOH gaps for patients, families, and caregivers.

To address heightened gaps in SDOH caused by societal closure, GWEP-FQHC partnerships launched food drives and delivered respite bags. Emergency housing information was made available for those at risk of homelessness. An urgent need arose to focus efforts on addressing loneliness and social isolation for patients and caregivers alike, and the disparities linked to loneliness in these populations were significant (Adepoju et al., 2021, 2022; Kotwal et al., 2021). GWEP-FQHC partnerships created programs to address social isolation. One partnership initiated a virtual friendly visitor program that paired patients with volunteers in the community for virtual weekly check-ins and conversations. Other partners developed student programs, which recruited medical, pharmacy, occupational therapy, physical therapy, social work, and nursing students to make regular phone calls to isolated seniors across the county.

## Impact/Lessons Learned

The dramatic differences in COVID-19 cases and mortality disparities by age and ethnicity were profoundly accentuated in the FQHC populations, especially for older adults. The FQHCs played a critical role in local disaster response to underserved and vulnerable older adults during the pandemic crisis. GWEP collaborations contributed to the FQHC responses in many ways, including continuing care for older adults, creating educational materials, helping to develop telehealth programs, and keeping age-friendly care concepts at the forefront of the partnerships.

Innovative GWEP-FQHC collaborations during the pandemic enabled partnerships to begin addressing workforce shortages, telehealth literacy and access, and exacerbated care gaps. These partnerships are uniquely positioned to work towards preparing our safety net systems to address the needs of a burgeoning underserved geriatric population. Though older adults currently comprise less than 10% of the total FQHC population, the trend over time has shown a year-to-year increase. As forged in the pandemic, the flexibility and adaptability gained by GWEP-FQHC partnerships can reduce health inequalities for the growing population of vulnerable and diverse older adults now and in the future.
